# Effects of Transfer from Breeding to Research Facility on the Welfare of Rats

**DOI:** 10.3390/ani4040712

**Published:** 2014-12-03

**Authors:** Johanna W. M. Arts, Nynke R. Oosterhuis, Klaas Kramer, Frauke Ohl

**Affiliations:** 1 Department of Animals in Science & Society, Division of Animal Welfare & Laboratory Animal Science, Veterinary Faculty, Utrecht University, Utrecht, 3584 CM, The Netherlands; E-Mails: n.oosterhuis@umcu.nl (N.R.O.); k.kramer@vu.nl (K.K.); f.ohl@uu.nl (F.O.); 2 Harlan Laboratories B.V., P.O. Box 553, Venray, 5800 AN, The Netherlands; 3 Department of Human and Animal Physiology, P.O. Box 338, Wageningen University and Research Centre, Wageningen, 6700 AH, The Netherlands; 4 Department of Occupational Health, Safety and Environment, Free University, Amsterdam, 1081 HV, The Netherlands

**Keywords:** transportation, transfer, body temperature, ambient temperature, glucose, corticosterone, behavior, Thermochron iButton, rats

## Abstract

**Simple Summary:**

This study measured effects of transfer on body temperature, stress hormone levels, body weight, behavior and water and food intake in rats. Environmental temperature strongly affected body temperature of rats and needs to be controlled. Male rats need to habituate for at least one week, females for two weeks after transfer.

**Abstract:**

Transfer from the breeding facility to a research facility is a stressful event for laboratory animals. Heat stress has been reported to constitute one of the major concerns during transport of animals. This study measured ambient and body temperature, corticosterone and glucose levels, body weight, behavior and water and food intake before, during and after transfer in Wistar rats. Decreased body weight, water and food intake were observed on the day of transfer in rats. Environmental temperature strongly affected body temperature of rats and needs to be controlled. Male rats need to habituate for at least one week, females for two weeks after transfer.

## 1. Introduction

The transfer of laboratory animals from the breeding facility to research facilities embraces a variety of challenges for the animals, such as physiological and social stress due to handling, with new sounds and smells, fluctuations in temperature and humidity, disruption of light-dark cycle, separation from cage mates, and new caretakers [1,2]. Such stressors lead to activation of the hypophysal-pituitary-adrenal (HPA)-axis, promoting secretion of corticosterone and production of glucose (for review see [3]). Several studies on transportation of rodents report an increase in plasma corticosterone [2,4,5,6,7] and plasma glucose levels [8], as well as disrupted behavior [2,6,9], though occasionally a decrease in corticosterone and glucose levels in transported rats have been found [10]. Also, decreased heart rate, blood pressure, and body weight have been related to the process of transportation in rodents [2,9,11] and a decrease in glucose levels was detected in rabbits transported to the slaughter house [12].

Heat stress has been reported to constitute the most frequently occurring cause of death in transported animals and thus might be considered as one of the major stressors during transfer [13]. A previous study on transport in rats showed highly variable temperatures in transportation boxes [14]. During a pilot study with mice at Harlan Laboratories BV (The Netherlands), the peak temperature inside the transportation box was found to exceed 30 °C [15]. Syversen *et al.* (2008) for example found fluctuations in temperature of 11 °C during air transfer in more than 60% of shipments [16]. It is known that stress-induced-hyperthermia and ambient temperature (T_a_) affect body temperature (T_b_) [17,18,19,20,21,22]. Results of studies measuring T_b_ before and after transfer, however, are inconsistent: Capdevilla *et al.* (2007) [11] found no effect of transfer of rats on T_b_, while Dallman *et al.* (2006) [23] observed an increase in T_b_ of rats when moving a cage within the same room or to another room, respectively. Stemkens-Stevens *et al.* (2009) transported guinea pigs for a shorter or longer period, respectively, which resulted in a decreased T_b_ in both conditions [9]. Changes in T_b_ have been shown to affect physiological parameters [24,25,26], metabolism and behavior [19], and plasma corticosterone and glucose [27].

In laboratory animal vivaria either a constant temperature within the rat’s thermal neutral zone is used or suitable cage enrichment is supplied in order to give the animals the opportunity of self-regulating their environmental temperature. Neither a constant temperature, nor cage enrichment is present during transfer. It is to be expected that fluctuations in T_a_ during transfer will modulate T_b_ in laboratory animals during and after transfer, and that transfer stress in combination with ambient temperature fluctuations may result in either hypo- or hyperthermia in transported animals. Both short termed modulation in ambient temperature and potential hyperthermia may constitute a serious challenge for the adaptive capacities for animals that are being transferred from a breeder to a research facility. Before transfer, animals are housed in stable environmental conditions, which are optimized for temperature, humidity, and air composition. During transfer, animals can experience temperatures that fluctuate and deviate from the temperatures they are used to, and after transfer animals need to adapt to new standard conditions. In this study, we measured T_a_ and T_b_ and their interaction before, during, and after transfer to elucidate the animals’ responses to such temperature challenges, which no other study has measured so far. How temperature affects the animal during and after transfer was measured biochemically, by plasma corticosterone and glucose.

The transfer of research animals withholds a multitude of procedures, which results in a multifactorial stressor that can hardly be disentangled. Therefore, the transfer procedure as referred to in our studies embraces weighing and packing of the animals, multiple movements of the transportation boxes between buildings/areas (barrier-holding area-logistic center–research facility-in house) and includes the change of environment and caretakers.

The present study extends on previous findings on the effect of transfer procedures in male and female rats by now relating effects to the animals’ core temperature, to body weight, body temperature, food and water intake, plasma-corticosterone and -glucose (indicating hormonal stress response) and home cage behavior [14].

## 2. Materials and Methods

Experimental procedures were approved by the Animal Ethics Committee of Utrecht University.

### 2.1. Animals and Housing

Immediately after weaning 48 male (aged 3 weeks, weighing 38.5 ± 2.5 g) and 48 female (aged 3 weeks, weighing 37.8 ± 2.5 g) Wistar rats (HsdCpb:WU, Harlan Laboratories BV, The Netherlands) were moved in house from the animal breeding room to the animal room of the Surgical Unit of Harlan NL. All rats were color coded on their backs with spray for individual recognition, which was repeated weekly after weighing. Rats were housed in groups of three in translucent type IV Makrolon cages (Floor area 1815 cm^2^) with bedding (wood shavings), an opaque black polyethylene tube, and tissues. The rats had free access to processed water (acidified (pH 5.8–6.4), chlorinated (6–8 ppm), softened and filtered (0.02 microns)) and food (Harlan Teklad 2018S, irradiated Global 18% protein rodent diet, Harlan, Madison, WI, USA). The rats were kept under a 12 h:12 h light regime, lights on at 6.00 am, in a temperature (22–24.5 °C) and humidity (50%) controlled vivarium. Group compositions of the rats remained constant during housing and during and after transfer.

### 2.2. Experimental Setup

Three days after weaning, a temperature logger was implanted in one rat per cage to record core body temperature according to a randomized schedule. After a recovery period of 14 days, baseline measurements started. Four weeks after surgery, 48 rats (24 male and 24 female, eight cages per sex) were randomly selected and transferred to a new facility at the Utrecht University, the remaining 48 rats stayed at the breeding facility as a non-transferred control group. Housing conditions (temperature and humidity settings) were similar at both facilities (Harlan Laboratories BV and the Central Laboratory Animal Research Facility (CLARF) of the Utrecht University and Medical Centre).

The first week after surgery and the first week after transfer, body weight (BW) was registered daily in all animals. The remaining time all animals were weighed three times a week. Every week a blood sample was taken by tail vein incision as described in Paragraph 2.6.

### 2.3. Implantation Temperature Logger

Before implantation, temperature loggers (Thermochron iButton, DS1921L-F50, Dallas Semiconductor, Sunnyvale, CA, USA: Ø 17 mm, height 6 mm, weight 4 g) were programmed to start measuring simultaneously two weeks after implantation and to record and store T_b_ every 30 min. The iButtons were coated with a thin layer of silicon (Elastostil E41, Wacker) for water protection and applicability for abdominal use, and equipped with non-absorbable ligatures (Silkam 5-0, B.Braun, Melsungen, Germany) for fixation to the abdominal wall. The abdomen of fully anesthetized rats (Isoflurane 2.5%, O_2_:NO_2_ (1:2)) was shaved and disinfected. An incision was made to open the skin and abdominal wall. iButtons were attached to the ventrolateral part of the abdominal wall and the abdomen and skin were closed with absorbable sutures (Vicryl 5-0, Ethicon, San Angelo, TX, USA). Ten minutes before and one day after surgery, meloxicam (0.55 mg/kg, Metacam 5 mg/mL, Boehringer Ingelheim Vetmedica, St. Joseph, IN, USA) was administered subcutaneously (sc) to provide analgesia. Also, the rats received an antibiotic (5.55 mg/kg, Enrofloxacin 50 mg/mL, Bayer AG, Barmen, Germany) 10 min before surgery.

### 2.4. Transfer

The day before transfer (DBT1), the rats in the selected cages were packed with three animals per standard transportation boxes (62 × 44 × 15 cm), 1 cage per box. Besides bedding and sufficient food pellets from the home cage, a water source (HydroGel, Clear H2O, Portland, ME, USA) was included in the transportation box. The remaining cages of male and female rats stayed in the original animal room at the breeding facility.

In 13 transportation boxes, a temperature logger (Keytag, Askey Dataloggers B.V., Leiderdorp, The Netherlands) protected by wire mesh was attached to the wall of the box. To the outside of one box an iButton was attached to measure outside-box temperature. Immediately after packing, transportation boxes were moved to a temperature controlled holding area at the breeding site for approximately 100 min at a temperature of 22–25 °C. At the end of the afternoon, approximately 5 h after packing, rats were transferred by truck to a climate controlled logistic center (Boxmeer, The Netherlands), approximately 20 min and 27 km away, where the rats stayed overnight for 13 h at a temperature of T_a_ 17 °C, until transfer early the next morning (DAT0). Transfer from Harlan NL to CLARF Utrecht took six hours, with several stops for unloading, in a climate controlled van preset at 15 °C. All transfer procedures were performed according to work instructions of the breeding facility, which are based on the ILAR guidelines by Baldwin *et al.* (2006) [13]. In the new animal facility rats were exposed to unfamiliar caretakers, sounds, new cages, and a humidity higher by about 15%. Room temperature, water, food, and cage enrichment were equal to control situation.

### 2.5. Water and Food Intake

Food and water intake were weighed daily per cage in transported and control groups during and four days after transfer.

### 2.6. Blood Collection and Analysis

Blood was collected by tail incision [28] (approximately 200 μL/sample) a week before, directly after and weekly after transfer (five times total), between 3 h and 4 h after lights were switched on. Sampling of blood was the day’s first procedure. Blood samples were taken from two rats per cage (one rat with and one without a temperature logger), according to a randomized schedule with equal distribution over the animals. After sampling, animals were rewarded with a small amount of birdseed. Plasma-corticosterone (CORT) levels were determined in duplicate with radio immunoassay (RIA) specified for rats and mice (ImmuChem^TM^ double antibody ^125^I RIA kit, MP Biomedicals, LLC, OH, USA) according to the protocol of the supplier. Plasma-glucose (GLUC) was measured with the Glucose Assay Kit (BioChain, Hayward, WI, USA) following the o-Toluidine method [29] and read out using a microplate reader (DTX 880 Multimode Detector, Beckman Coulter Inc., Brea, CA, USA).

### 2.7. Behavioral Observations

All behavioral observations were scored live in the home cage in randomized cage order, directly after cage cleaning. Cleaning disturbs the animals and increases the level of activity in rats, which offers an opportunity to observe active behavior during the daylight period [30,31]. Observations were performed by continuous focal sampling for 5 min, 3–5 h after the start of the light period. One animal per cage was scored, according to a cage randomization schedule, with randomized equal distribution between animals with and without an iButton. Eighteen behavioral parameters were scored using the software The Observer (Noldus, Wageningen, The Netherlands) see table in Appendix. For analysis, the following three behavioral categories, each consisting of different behavioral elements, were used:

(1) Activity/Locomotion (LOC): consisting of the behaviors explore, rear, walk, shake, scratch, scan, and hop/jump.
(2) Social interactions (SI): consisting of social exploration, social groom, follow/chase, push and pin.
(3) Self grooming (GRO), consisting of six behavioral elements which together compose the cephalo-caudal-sequence that animals perform in an undisturbed situation: fore-paw licking, face/nose wash, head wash, body wash/fur licking, hind leg licking, tail/genitals licking [32].

### 2.8. Statistical Analysis

In the present study the cage was the experimental and therefore statistical unit [33]. If applicable, parameters were averaged per cage. Body weight data was corrected for weight of the iButton. Data, residuals, and variance were tested for Gaussianity using a One-sample Kolmogorov-Smirnov test. Having a temperature logger was found not to have a significant effect and was therefore left out as factor. To detect overall effects and differences between before and after transfer for all parameters apart from behavior, a Repeated Measures ANOVA was executed. Data was considered significant when *p* < 0.050. An Independent-Samples *t*-test was used to compare transfer *vs.* control cages and male *vs.* female with each other per day for T_b_, BW, food and water intake, CORT and GLUC. Alpha was corrected for multiple factors using the Dunn-Šidák correction and data was considered significant when *p* < 0.025. Pearson’s correlation coefficient was calculated for the correlation between T_a_ and T_b_.

Behavior was analyzed using a Linear Mixed Model, obtaining the best fit using a model with a random intercept. Data was analyzed both split and un-split for treatment, with factors treatment, time/observation, sex and their interactions, all non-significant interaction terms were removed. Dependent variables were the duration percentages of total grooming (=sum of all self-grooming behaviors), total social interaction behavior and total active and locomotor behavior. The Q-Q plots of the residuals indicated that all behavioral variables were normally distributed.

Some plasma samples had CORT values below the detection limit (7.7 ng/mL). These data values were replaced by the value pre-half of the detection limit [34].

Calculations were performed using Microsoft Excel 2003. SPSS 16 (IBM SPSS Statistics, New York, NY, USA) was used for statistical analysis. All data is displayed as mean ± SD, both in tables and figures.

## 3. Results

### 3.1. Body Weight

**Figure 1:**
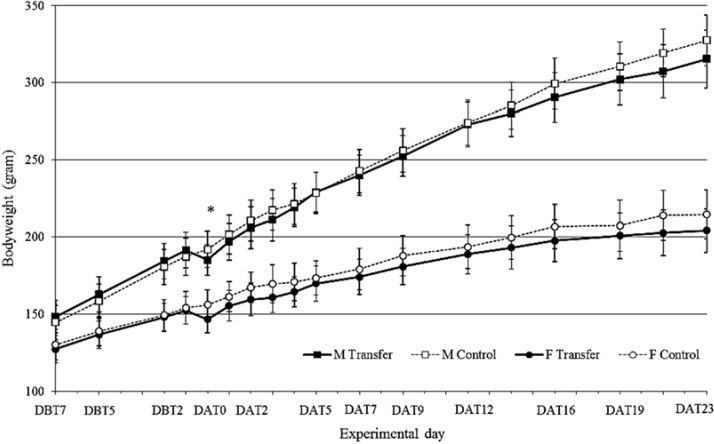
Body Weight (gram ± standard deviation (SD)) in male (squares) and female (circles), transferred (black symbols-solid line) and control (white symbols-dotted line) rats, 7 days before (DBT) until 23 days after (DAT) transfer (*n* = 8). * indicates significant different growth between transferred (TF) and control (CO) individuals.

There was a sex effect on bodyweight (M > F, F_(1,28)_ = 540.0, *p* < 0.001) (Figure 1). Transfer resulted in a decrease in BW of transferred animals compared to the control animals. This difference was significant at day of transfer (male: t_(14)_ = −2.63, *p* = 0.020, female: t_(14)_ = −2.68, *p* = 0.018). One day after transfer this decrease was no longer significant. In the week before transfer, males increased in weight 7.1 ± 1.5 g/day. On the day of transfer they lost on average 6.2 ± 3.8 g (=3.2%), followed by an increased bodyweight gain of 11.8 ± 4.7 g the day after transfer. Females increased in weight the week before transfer 4.3 ± 1.4 g/day. On the day of transfer they lost on average 5.8 ± 2.6 g (=3.8%), followed by an increased bodyweight gain of 8.7 ± 3.8 g the day after transfer.

### 3.2. Water and Food Intake

Food and water intake are shown in Table 1. Over the total measured period there was a treatment effect (Transfer < Control: F_(1,28)_ = 36.63, *p* < 0.001), a sex effect (M > F; F_(1,28)_ = 69.59, *p* ≤ 0.001) and a time × treatment interaction (F_(3,85)_ = 73.36, *p* ≤ 0.001) found for water intake. During transfer, a water source was added to the transportation box. Less of this water source was consumed by the transferred animals, both male and female, compared to water consumed by male and female control rats (male: t_(14)_ = −9.47, *p* < 0.001, female: t_(14)_ = −12.30, *p* < 0.001) during the same period. Compared to male transferred rats, male control rats showed a higher water consumption at day 3 (t_(14)_ = −2.98), *p* = 0,01) and Day 4 after transfer (t_(14)_ = −7.94, *p* < 0,001). Water intake of female transferred rats was significantly higher at day 1 (t_(14)_ = 3.80, *p* ≤ 0.002) and in female control rats on Day 4 (t_(14)_ = −4.03, *p* = 0,001). Over the total measured period, there was a time × sex interaction (F_(4,106)_ = 2.50, *p* = 0.050), a time × treatment interaction (F_(4,106)_ = 172.02, *p* < 0.001) and a time × sex × treatment interaction (F_(4,106)_ = 3.61, *p* = 0.010) found for food intake. Food intake was reduced during transfer in both male and female transferred rats (male: t_(14)_ = −11.83, *p* < 0.001, female Z = −3.37, *p* < 0.001). Food intake was increased in transferred female rats for three days after transfer and in transferred male rats for two days after transfer (*p* ≤ 0.010), compared to control animals.

**Table 1:** Water and food intake (mean ± SD grams) per cage of transferred and control rats (M = male and F = female). Day 0 is day of transfer. * Significantly different from control per *t*-test (*p* < 0.025) between transfer and control group, *n* = 8 cages of three animals.

**Water (g)**	**Day 0**	**Day 1**	**Day 2**	**Day 3**	**Day 4**
**M Transfer**	42.13 ± 11.10 *	80.75 ± 18.43	66.38 ± 10.18	71.50 ± 10.54 *	71.38 ± 5.07 *
**M Control**	86.63 ± 7.31	73.63 ± 6.61	76.13 ± 7.62	85.63 ± 8.48	91.38 ± 5.01
**F Transfer**	26.63 ± 6.07 *	66.75 ± 5.63 *	50.13 ± 3.83	52.38 ± 4.60	52.38 ± 5.45 *
**F Control**	64.13 ± 6.13	55.13 ± 6.58	56.63 ± 6.80	61.63 ± 10.49	68.38 ± 9.81
**Food (g)**	**Day 0**	**Day 1**	**Day 2**	**Day 3**	**Day 4**
**M Transfer**	40.63 ± 6.02 *	65.25 ± 7.57 *	60.63 ± 5.29 *	61.88 ± 6.31	61.00 ± 3.96
**M Control**	69.50 ± 3.38	44.13 ± 2.59	54.00 ± 3.42	56.75 ± 2.76	60.50 ± 2.88
**F Transfer**	31.25 ± 3.28 *	53.50 ± 3.16 *	45.75 ± 3.49 *	45.75 ± 3.54 *	44.63 ± 3.70
**F Control**	50.75 ± 4.83	33.13 ± 3.64	39.75 ± 4.06	39.13 ± 4.09	47.00 ± 3.30

### 3.3. Body Temperature

*Post mortem* examination after the completion of the study showed that most of the iButtons were attached to the abdominal wall with one suture. Two iButtons were encapsulated, one of which was detached and found back at the opposite side of the abdomen. However, the two concerning rats had shown no clinical signs. After data analysis of the loggers it was determined that it was not necessary to remove them from the study.

**Figure 2:**
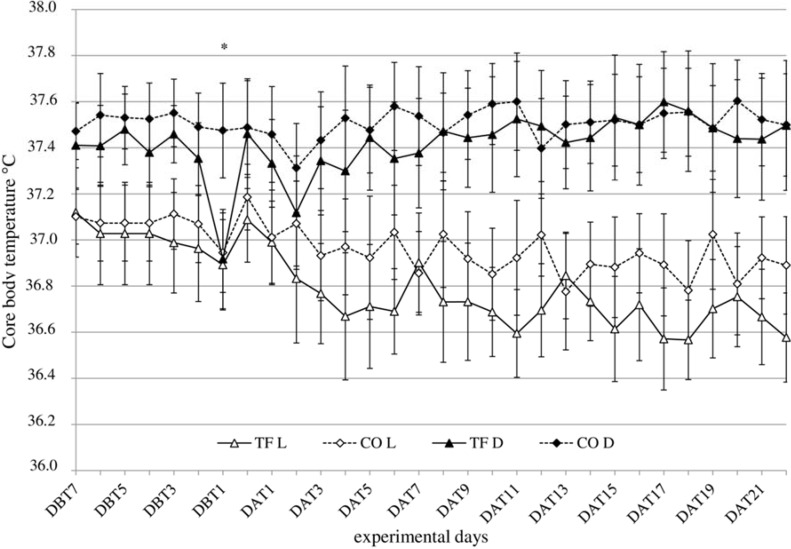
Core body temperature T_b_ (°C ± SD) in transferred (TF: triangles, solid line) and control (CO: diamonds, dotted line) rats during dark (black symbols) and light (white symbols) period, 7 days before (DBT) until 22 days after (DAT) transfer (*n* = 16), sexes pooled. * indicates significant difference between TF and CO.

No sex effect was found for body temperature T_b_, therefore data was pooled for analysis and the results are shown in Figure 2. During dark periods, T_b_ was increased for all groups compared to light periods, shown as a light period effect (F_(1,52)_ = 160.50, *p* < 0.001), except on the day before transfer (DBT1) for transferred animals (Figure 2), when transferred rats were packed in transportation boxes and were transferred to the logistic center awaiting final transfer to the CLARF. During the dark period there was a before-after × treatment interaction (F_(1,26)_ = 9.82, *p* = 0.004), which was not found during the light period. During the dark period of transfer a decreased T_b_ was found in both sexes (t_(13)_ = −4.77, *p* = 0.001).

### 3.4. Ambient Temperature

**Figure 3:**
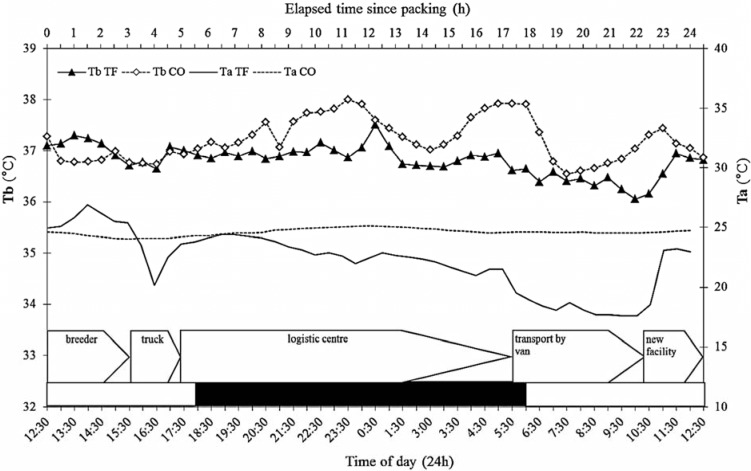
Core body temperature T_b_ of transferred (triangles) and control (diamonds) rats and ambient temperature T_a_ inside transportation boxes (solid line) (°C ± SD) during transfer and in control area (dotted line) (top x-axis: time since packing, bottom x-axis: actual time). The black-white bar at bottom indicates light (white) and dark (black) periods. Different stages during transfer are included. *n* = 16, sexes pooled.

The highest T_a_’s inside the transportation boxes were measured when transportation boxes were collected at the holding area of Harlan NL (male: 28.50 °C, female: 27.40 °C, Figure 3). Environmental temperature varied between 22 °C and 25 °C at the holding area. During transfer to CLARF Utrecht with the climate controlled van (15 °C), T_a_ inside transportation boxes with male rats decreased to 17.30 °C and 17.10 °C for female rats. During the period of transfer T_a_ at the housing facility of control groups varied between 24.1 °C and 25.1 °C.

A significant correlation was detected between T_a_ (solid line) and T_b_ (solid line with diamonds) during transfer of rats (Figure 3, *r* > 0.7, *p* < 0.001). In the control situation T_a_ (dotted line) and T_b_ (grey line with circles) were not correlated (r = −0.061, *p* > 0.050).

### 3.5. Blood Parameters

**Figure 4:**
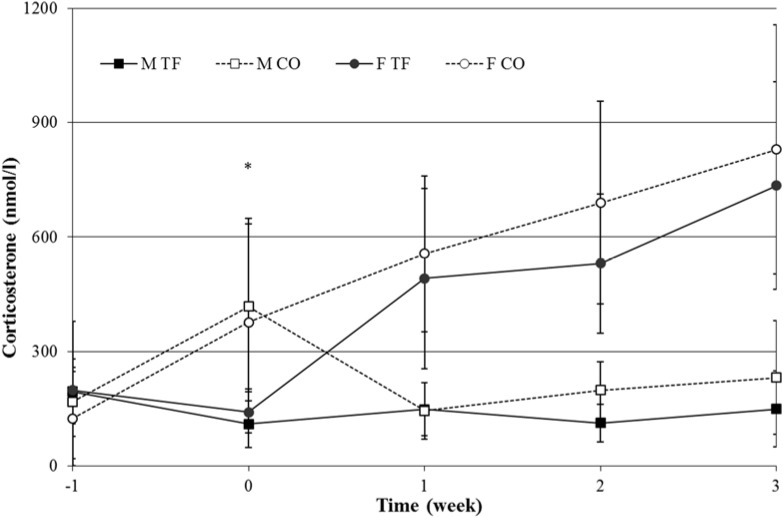
Plasma corticosterone levels (nmol/L) in male (squares) and female (circles), transferred (black symbols-solid line) and control (white symbols-dotted line) rats, one week before, on day of transfer and one, two and three weeks after transfer (*n* = 8). * indicates significant difference between TF and CO.

In CORT levels a time × sex interaction (F_(4,112)_ = 21.14, *p* < 0.001) and a time × treatment interaction (F_(4,112)_ = 3.93, *p* = 0.005) were found (Figure 4). Female rats had higher CORT levels that also increased more over time than male rats (F_(1,28)_ = 80.96, *p* < 0.001).

**Figure 5:**
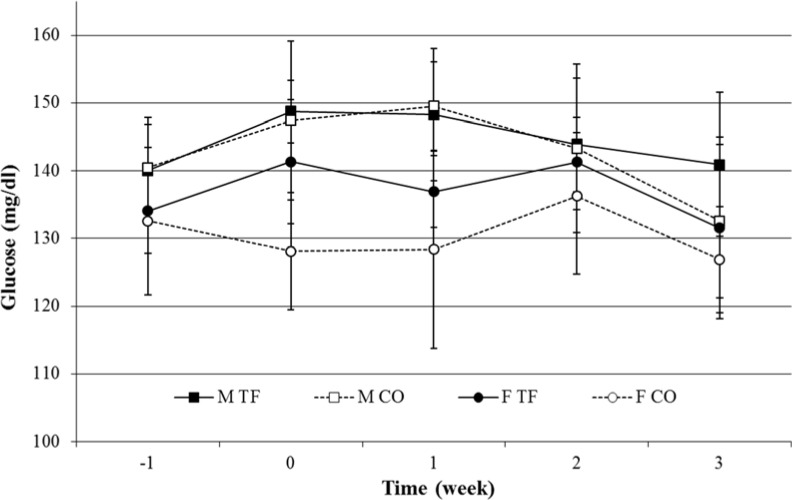
Plasma glucose levels (mg/dL) in male (squares) and female (circles), transferred (black symbols-solid line) and control (white symbols-dotted line) rats, one week before, on day of transfer and one, two and three weeks after transfer (*n* = 8).

GLUC levels of female rats were lower than GLUC levels in male rats (F_(1,27)_ = 23.19, *p* < 0.001). A time × sex (F_(4,108)_ = 4.71, *p* = 0.002) effect was found, but no transfer effect (Figure 5).

### 3.6. Behavior

Analysis of the behavioral data showed a time × sex interaction on all parameters (Table 2). Further analysis of the behavioral data (Table 3) showed that in female rats transfer decreased social behavior and increased grooming behaviors. Activity was increased 1, 2 and 3 weeks after transfer in all groups. Social behavior was decreased after transfer in all groups, except in female control rats 1 week after transfer. Grooming was increased in female transferred rats directly after unpacking, but differed no longer from baseline after one week of acclimatization. After 3 weeks of acclimatization all behavioral elements in female rats seemed to have stabilized. In male rats differences in behavioral elements were found comparing after-transfer observations with baseline, but no significant differences were found between observations after transfer.

**Table 2:** Behavioral observations; Effects (bold *p*-values) and trends (italic *p*-values) of transfer on the behavioral categories Locomotor activity (ACT), Social behavior (SOC) and Grooming (GRO) *n* = eight cages.

Effect	ACT	SOC	GRO_tot_
Sex	F_(32,32)_ = 5.73, *p* = 0.023	NS	NS
Treatment	NS	NS	NS
Observation (time)	F_(88,32)_ = 12.29, *p* < **0.001**	F_(110,30)_ = 9.00, *p* < **0.001**	F_(103,104)_ = 4.49, *p* = **0.005**
Sex x Treatment	NS	NS	NS
Sex x time	F_(88,32)_ = 4.00, *p* = **0.005**	F_(110,30)_ = 3.08, *p* = **0.019**	F_(94,104)_ = 4.70, *p* = **0.002**
Treatment x time	NS	NS	NS
Sex x Treatment x time	F_(90,32)_ = 2.35, *p* = 0.077	F_(110,30)_ = 2.46, *p* = 0.066	NS

**Table 3:** Behavioral observations; Comparison between observations, −1: 1 week before transfer; 0: directly after transfer; 1: 1 week after transfer; 2: 2 weeks after transfer; 3: 3 weeks after transfer (significant: bold *p*-value, trend: italic *p*-value), Split for sex. ↑: increase or ↓: decrease of behavior during obseravtion on vertical axis (**left**) compared to behavior during observation on horizontal axis (**top**) *n* = eight cages.

Obs		−1			0			1			2		
	sex	ACT	SOC	GRO	ACT	SOC	GRO	ACT	SOC	GRO	ACT	SOC	GRO
0	F	NS	↓**0.003**	↑<**0.001**									
	M	↑*0.043*	↓*0.052*	NS									
1	F	NS	NS	NS	NS	↑*0.007*	↓<**0.001**						
	M	↑<**0.001**	↓**0.002**	↑*0.017*	NS	NS	NS						
2	F	↑**0.002**	↓*0.011*	NS	↑**0.004**	NS	↓<**0.001**	↑**0.003**	↓*0.024*	NS			
	M	↑<**0.001**	↓**0.003**	↑*0.013*	NS	NS	NS	NS	NS	NS			
3	F	↑<**0.001**	↓<**0.001**	NS	↑<**0.001**	NS	↓<**0.001**	↑<**0.001**	↓**0.001**	NS	NS	NS	NS
	M	↑<**0.001**	↓**0.001**	↑**0.004**	NS	NS	↓*0.056*	NS	NS	NS	NS	NS	NS

(Dunn-Šidák correction for multiple comparison(per sex): 1−((0,95)^(1/*n*)), *n* = 10 (number of comparisons) α = 0, 005).

## 4. Discussion

The present study investigated whether changes in core temperature of Wistar rats may be an underlying cause for findings in previous studies in which we found that transfer affected rats at both the physiological and behavioral level [2,14]. Further, in the current study we identified sex specific changes, which showed allostatic stabilization within 2 weeks, though inter-individual variability proved to be high [14]. Though hyperthermia was expected due to a combination of stress and elevated ambient temperature, this effect was not observed during or after transfer. This seems noteworthy, especially since the day before transfer, when animals were packed, was a warm summer day and temperature inside transportation boxes reached up to 28 °C.

Body temperature was recorded every 30 min in the present study. From the literature it is known that maximal rise of body temperature induced by disturbance was detected after 10 min in male NMRI mice [35], followed by a gradual decrease during the following 30–60 min [23,27]. While we did not find any significant increase of body temperature in rats, ambient and body temperature appeared to be significantly correlated during transfer, but not during the control phase. From these results we conclude that, at least under transfer conditions as used in our study, core temperature is unlikely to be the primary cause for physiological or behavioral transfer effects.

As body temperature is correlated with the animal’s overall activity [36], changes in both parameters are determined by circadian rhythm [37,38]. During transfer, however, body temperature appeared to be lower during the dark period in transferred rats when compared to the animals in the control situation, which may be explained by lower activity of the animals in the transportation box.

Further, after transfer, body temperature in transferred rats was lower during the light period when compared to control animals, which may be a response to differences in the management regime between facilities (e.g., fewer disturbances by personnel).

Transfer is a stressful event for rats, as shown in previous studies resulting in elevated levels of CORT [2,4,5,6,7]. Here, CORT levels in male rats of both experimental groups remained stable over time while all females revealed increasing CORT levels over the same time period. No significant transfer effect on CORT levels occurred, which contrasted previous findings. However, in extrapolating results from a study in rabbits where it has been shown that rewarding and handling at an early age led to more tame animals that also did not struggle during a tonic immobility test [39], we provided rats in the present study with a reward after blood sampling and weighing, which may have counteracted the stress caused by blood sampling, thus, resulting in less increase in plasma corticosterone levels compared to other studies. Except at baseline and directly after transfer, CORT levels of female rats were higher compared to male rats. It is known that the HPA-axis activity is sex-dependent, resulting in higher baseline CORT levels [39] and a more pronounced increase in CORT levels after exposure to a stressor in female rats [40]. Further, rats used in the present study were adolescent, implying the development of estrous in females, which also is known to increase CORT levels [41,42,43,44]. Similar to findings in the literature, decreased CORT levels in transferred female rats directly after transfer had been found in our previous study [14] and by Van Ruiven *et al.* (1998) [10]. Unexpectedly however, an increased level of CORT was measured in male control animals at the day of transfer, an effect that may be attributed to external conditions (here: in the replacement of a technician who collected the blood samples).

As result of a stress-response, the production of glucose (GLUC) has been reported to increase [45,46]. However, in the present study GLUC levels were within a normal range 134–219 mg/dL [47].

## 5. Conclusions

Bodyweight and food and water intake recovered within a few days after transfer. Behavior in this study shows time effects, most clearly in female rats, but no treatment effects, indicating that before-after transfer differences may be a matter of aging rather than of transfer-stress. Daily practice shows that most laboratory animals are transferred at an age at which they are still (physically) developing.

This study showed that it took one day for body weight and temperature, two days for water intake, and four days for food intake to return to control levels. Levels of CORT and GLUC were not affected by transfer in this study. Behavioral parameters indicate that female rats seemed to stabilize after two weeks of acclimatization, male rats within one week. Although core temperature in this study was not affected by transfer, it is recommended to keep environmental temperature and temperature inside transportation boxes as constant as possible, and within the physiologically neutral range of the animals, as external temperature strongly correlates with core body temperature in rats. We conclude from these findings that it is advisable to use an acclimatization period of at least one week in male and two weeks in female Wistar rats.

## Appendix

**Table A1:** Details of ethogram used.

Behavior	Description	
Exploration	Animal is sniffing air, the sawdust or cage wall. Collection of behaviors like mobile exploration, sniffing, root/dig and gnaw/nibble, manipulating shelter.	state
	Rearing during exploration is scored as rearing	
Walk	Obvious forward movement of the animal, with as goal to bridge a distance. Is scored when more than two steps are made with the hind legs	state
Rest (lie&sit)	Resting behavior, lying, and sitting without sniffing or looking around in an alert state.	state
Eat	Taking in food	state
Shake	Shaking of the head or shaking the whole body	event
Scan	Horizontal rocking movement of the head, with the rest of the body still. If performed in a rearing posture this is scored as scan	state
Rear	Animal is exploring (sniffing, watching) with its fore paws lifted off the ground. Only standing on its hind legs, or supported by putting the forepaws on the cage wall. If an animal sits sniffing and lifts its forepaws off the ground, this is a rear (also if the animal does not stretch its body)	state
Hop/Jump	Hop: horizontal movement of the animal by little jumps	state
	Jump: the animal jumps on the spot	event
Freeze	Animal freezes and stops all movements	state
In shelter	Animal is (partially) in the shelter. If not visible in the shelter, this is still scored as shelter	state
Social exploration	Animal is sniffing/exploring cage mate	state
Follow/Chase	Animal is following/chasing a cage mate	state
	Animal is being followed by a cage mate	state
Push	Animal is pushing a cage mate with its nose or paw	event
	Animal is being pushed by a cage mate	event
Pin	Animal is pinning a cage mate on its back or side	state
	Animal is being pinned by a cage mate	
	Often followed by social groom	state
Social groom	Animal is licking a cage mate or nibbling the fur of a cage mate	state
	Animal is being licked-nibbled by a cage mate	state
